# Turtleheading the Tough Aortic Necks! A Novel Endovascular Method to Avoid Bird-beaking, Invagination, and Stent Migration in Thoracic Aortic Grafts

**DOI:** 10.7759/cureus.2731

**Published:** 2018-06-03

**Authors:** Preston Hood, Maharshi Patel, Amanda Johnson, John Pirris, Jerry Matteo

**Affiliations:** 1 Department of Interventional Radiology, University of Florida College of Medicine, Jacksonville, USA; 2 Cardiothoracic Surgery, University of Florida College of Medicine, Jacksonville, USA

**Keywords:** endovascular treatment of aneurysm, bird-beaking, invagination, stent-migration, thoracic aortic aneurysm, endovascular intervention, thoracic aortic graft, aortic aneurysm

## Abstract

Conventional repair of aortic pathology such as thoracic aortic aneurysms (TAA), aortic dissections, and intramural hematomas (IMH) involves major cardiothoracic surgery. Complication rates can be as high as 30%, therefore percutaneous endograft placement has become the new gold standard. However, not every patient is a suitable candidate for endovascular repair of a thoracic aneurysm, especially, patients with a very short proximal landing zone neck or a difficult type II or type III configuration of the aortic arch. Emerging techniques have been described in the literature, but until now none have been able to confidently conquer this problem. Stacked stents in a “turtlehead” fashion offer a solution to this obstacle. The turtlehead technique utilizes commercially available stents deployed in an on-label fashion to create a rigid yet conformable endograft that can precisely treat difficult proximal landing zone necks.

## Introduction

**Figure 1:**
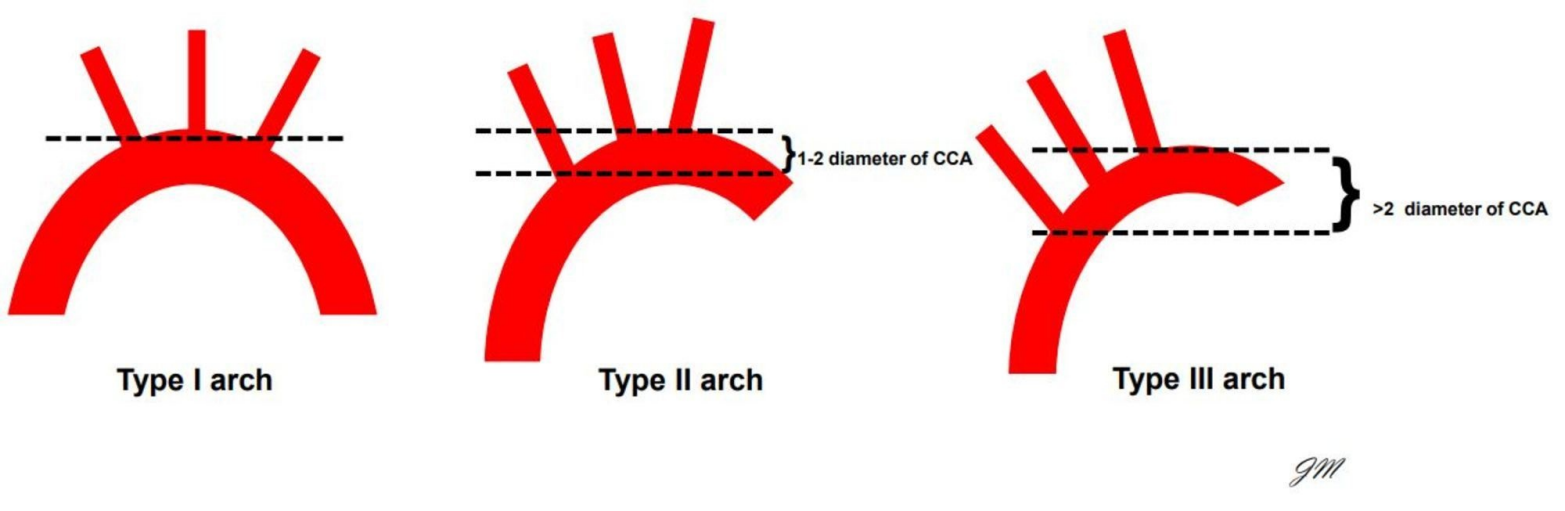
Shows a schematic of the configurations of the aortic arch. The complication of bird-beaking becomes more prevalent as the angulation within the arch increases from type II to type III.

Approximately 10% of thoracic aortic aneurysms (TAA) occur in the aortic arch and 40% occur in the descending aorta [1]. The majority of TAA are asymptomatic given the nature of their indolent course. The five-year survival rate of untreated TAA measuring at least 6 cm is approximately 54%, therefore treatment is imperative. The size of the aneurysm, the arch anatomy, and proximal landing zone length are important factors to be considered during endovascular aortic stent grafting for the repair of TAA. There are three different types of aortic arch which are delineated based on the vertical distance from the innominate artery and top of the aortic arch [2]. Type 1 arch is characterized by a distance of <1 diameter of the left common carotid artery (CCA). Type 2 vertical distance measures between 1-2 diameters of CCA, and type 3 represents diameters >2 (Figure 1).

Stent-grafts that do not conform to the contours of the aortic arch can extend into the lumen at the inner curvature of the arch, forming a bird-beak on imaging [3]. Patients with type 3 arch anatomy present a complex repair profile due to the concern of bird-beaking, invagination, and stent migration. Bird-beaking is a term that is used to describe when a stent does not have good wall apposition proximally allowing pressure and force on the outside of a stent instead of on the inner lumen. Bird-beaking can lead to invagination in which the proximal portion of the stent infolds on itself leading to abrupt narrowing or complete occlusion. Approximately 1.5 cm landing zone is needed for adequate positioning and covering of the aneurysm with an endovascular graft. If the aneurysm is in close proximity to the origin of the left subclavian artery, for adequate seal of the landing zone, coverage of the left subclavian artery must be performed. Coverage of the origin of the left subclavian artery for adequate proximal landing zone occurs in approximately 35% of cases [4-6]. This article is presented to illustrate a novel technique named “turtleheading” for the treatment of complex aortic pathology. The turtlehead technique is utilized for rigidity, conformability, and precision. Although the authors have successfully performed this technique on multiple patients, three demonstrative examples (aneurysm, dissection, and traumatic injury) of patients with difficult arch anatomy and short landing zones treated with endovascular graft repair using the turtlehead technique are shown.

Case 1 is an 89-year-old male with history of congestive heart failure, coronary heart disease, pulmonary emphysema, hypertension, and atrial fibrillation. A computed tomography angiography (CTA) of the chest was obtained which showed a massive thoracic aortic aneurysm measuring up to 9 cm in diameter and a type III arch with a short proximal landing zone neck. Endovascular aortic stent grafting with a single graft was not feasible in our case due to concern of migration and the possible complication of bird-beaking, which can lead to invagination and a disastrous outcome.

Case 2 is a 75-year-old female with history of hypertension, chronic obstructive pulmonary disease, lung cancer, and chronic pulmonary emboli presented with acute onset back pain. A CTA of the chest demonstrated a large intramural hematoma (dissection) from the level of the aberrant right subclavian artery to the celiac trunk.

Case 3 is a 61-year-old male with history of coronary artery disease and left-sided pacemaker. He was riding a bicycle and was struck by a vehicle. A trauma thoracic CTA was performed showing an aortic tear with pseudoaneurysm formation just distal to the left subclavian artery with only a 5.9 millimeter (mm) landing zone proximal neck along the lesser curvature of the aortic arch.

## Technical report

**Figure 2:**
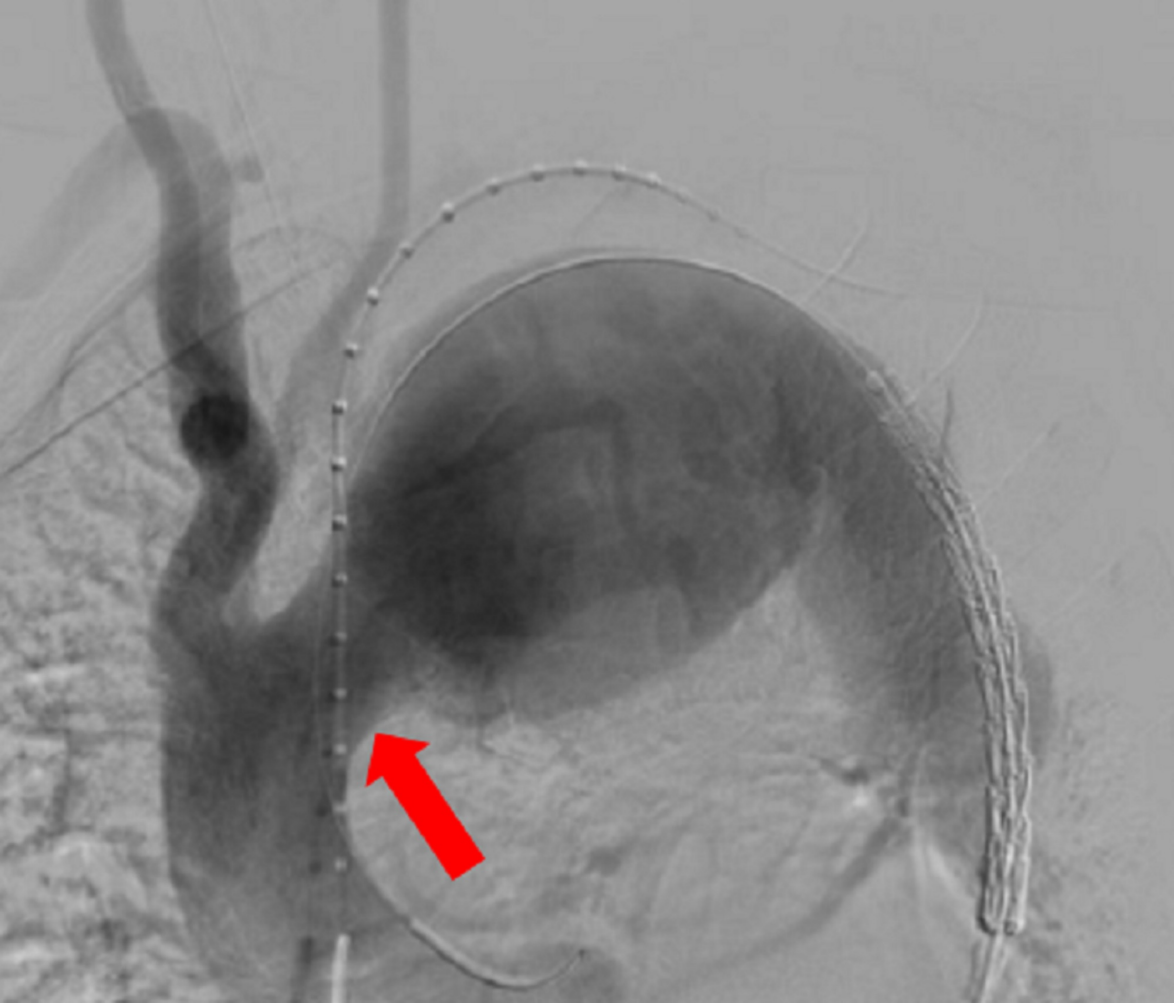
Angiogram was performed with a flush catheter from a left brachial approach showing a massive thoracic aortic aneurysm measuring 9 cm and a type III arch with a short proximal landing zone (red arrow) neck.

For the aneurysm case presented, percutaneous left brachial arterial access was performed using the micropuncture system and upgraded to a 6 French vascular sheath. Percutaneous right common femoral arterial access was performed using the micropuncture system after a groin cut down by the vascular surgical team and upgraded to a 24 French Dry Seal sheath (W L Gore and Associates, Flagstaff, AZ, USA). An angiogram was performed with a flush catheter from a left brachial approach showing a massive thoracic aortic aneurysm measuring 9 cm and a type III arch with a short proximal landing zone neck (Figure 2).

**Figure 3:**
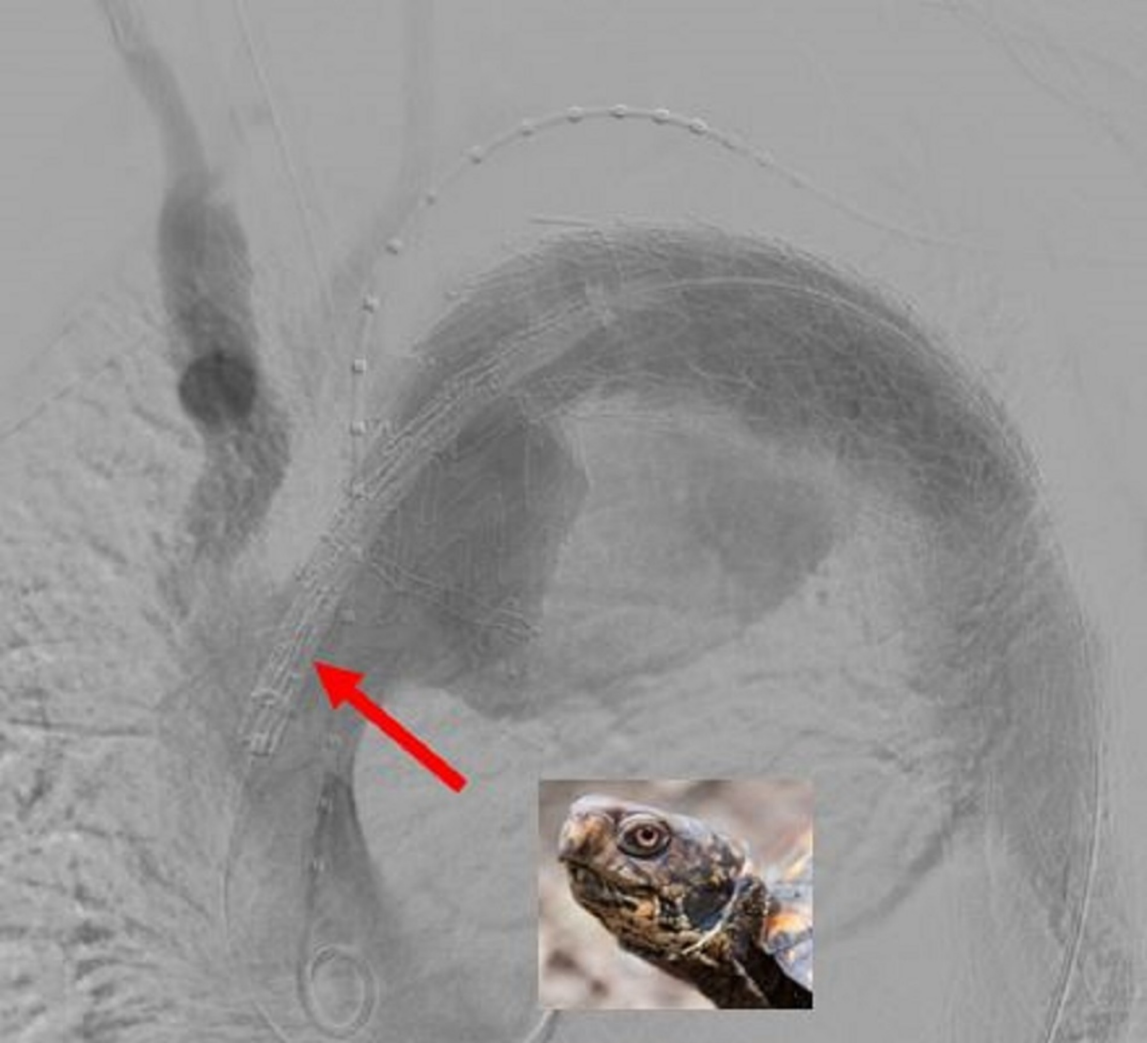
The final stent is placed with 85-90% overlap with only approximately a centimeter of stent deployed proximally for exact precision known as the turtlehead (red arrow).

Due to the descending thoracic aortic aneurysm size, location, and subsequent short landing zone, there is increased likelihood of bird-beaking and endoleak. Therefore, the decision was made to deploy multiple thoracic aortic stent grafts in a turtlehead overlapping fashion beginning distally to proximally. The distal portion of the aortic treatment zone measured 28 mm in diameter and the proximal portion of the native aorta measured 38 mm. Therefore, through the right common femoral sheath, five thoracic aortic stent/grafts [34 mm x 34 mm x 10 cm, 37 mm x 37 mm x 10 cm, 40 mm x 40 mm x 10 cm, 45 mm x 45 mm x 10 cm, and 45 mm x 45 mm x 10 cm] (W L Gore and Associates, Flagstaff, AZ, USA) were sequentially deployed in the aforementioned turtlehead overlapping fashion. The amount of overlap increases with each subsequent stent from distal to proximal. The final stent is placed with 85-90% overlap with only approximately a centimeter of stent deployed proximally for exact precision known as the turtlehead (Figure 3).

**Figure 4:**
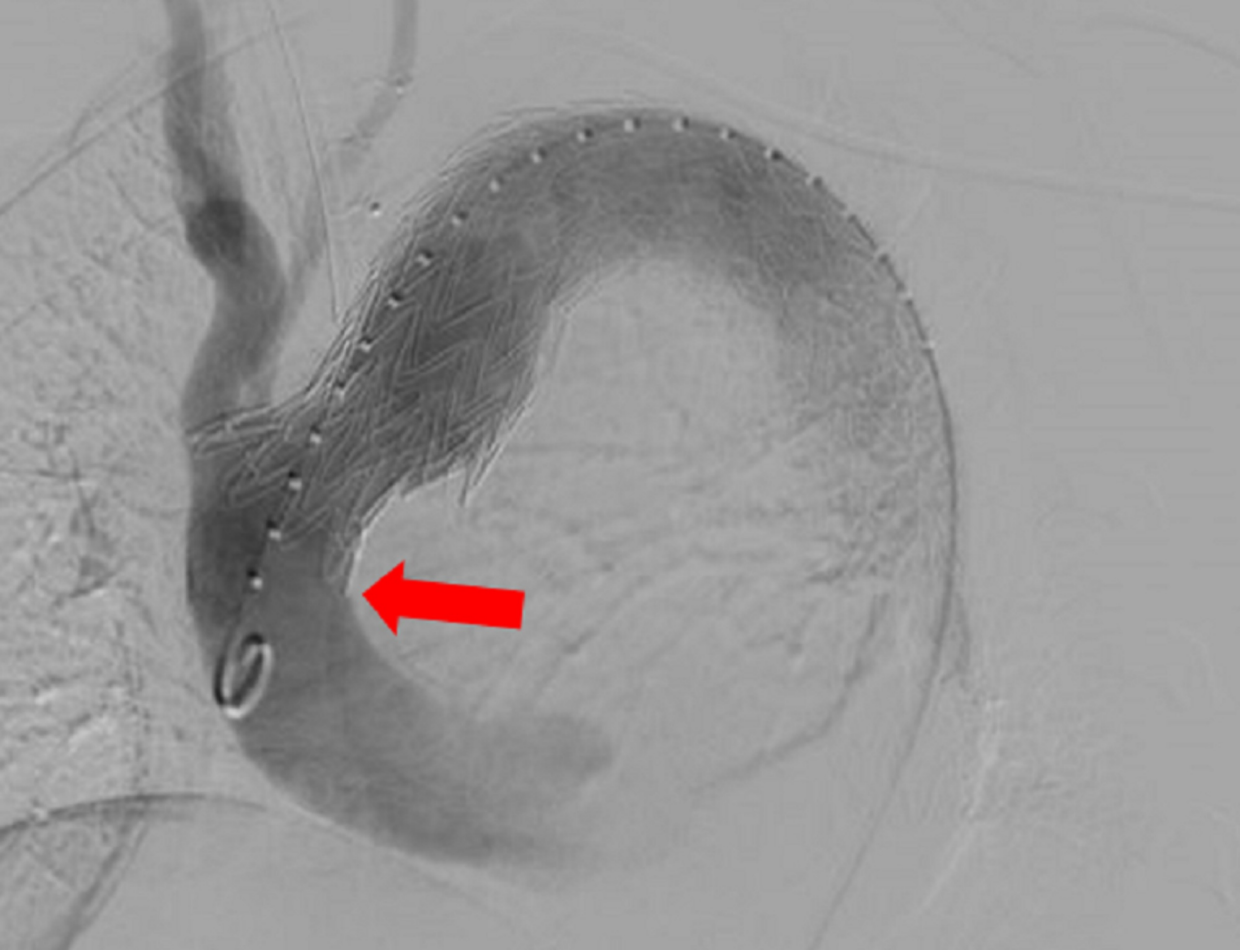
Aortography was then performed through pigtail flush catheter placed in the thoracic aortic arch which demonstrated complete coverage of the descending thoracic aortic aneurysm. No endoleak or bird-beaking identified (red arrow).

This technique provides rigidity to the stent grafts which improves accuracy of placement and decreased risk of movement of the final stent/graft placement, bird-beaking or endoleak. The final endograft was positioned proximal to the origin of the left subclavian artery and uneventfully deployed. Subsequently, balloon angioplasty with a trilobed balloon (W L Gore and Associates, Flagstaff, AZ, USA) was performed at the proximal and distal ends of the grafts. Aortography was then performed through pigtail flush catheter placed in the thoracic aortic arch which demonstrated complete coverage of the descending thoracic aortic aneurysm (Figure 4).

**Figure 5:**
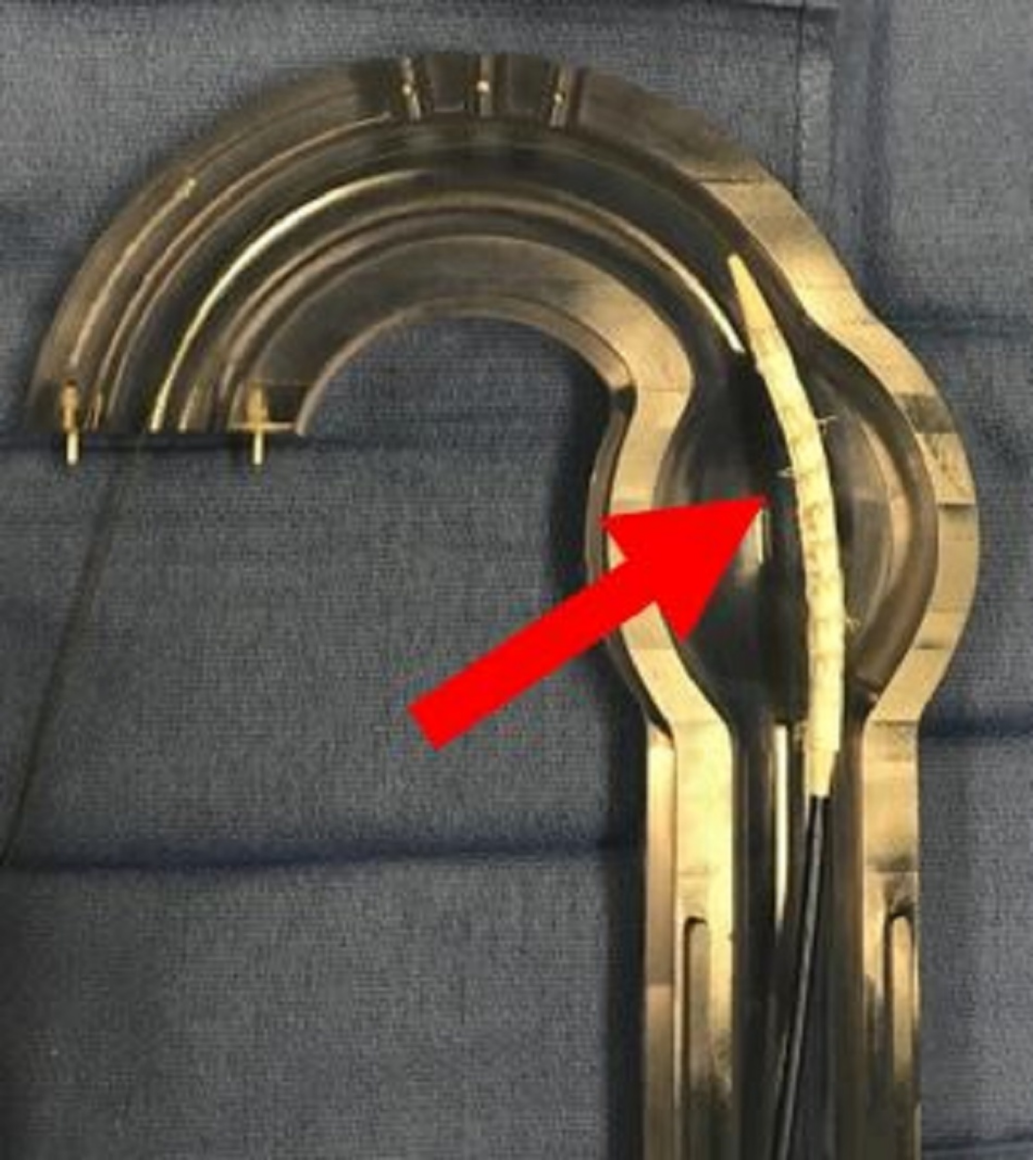
Aortic glass model with anchor thoracic aortic graft in place (red arrow).

**Figure 6:**
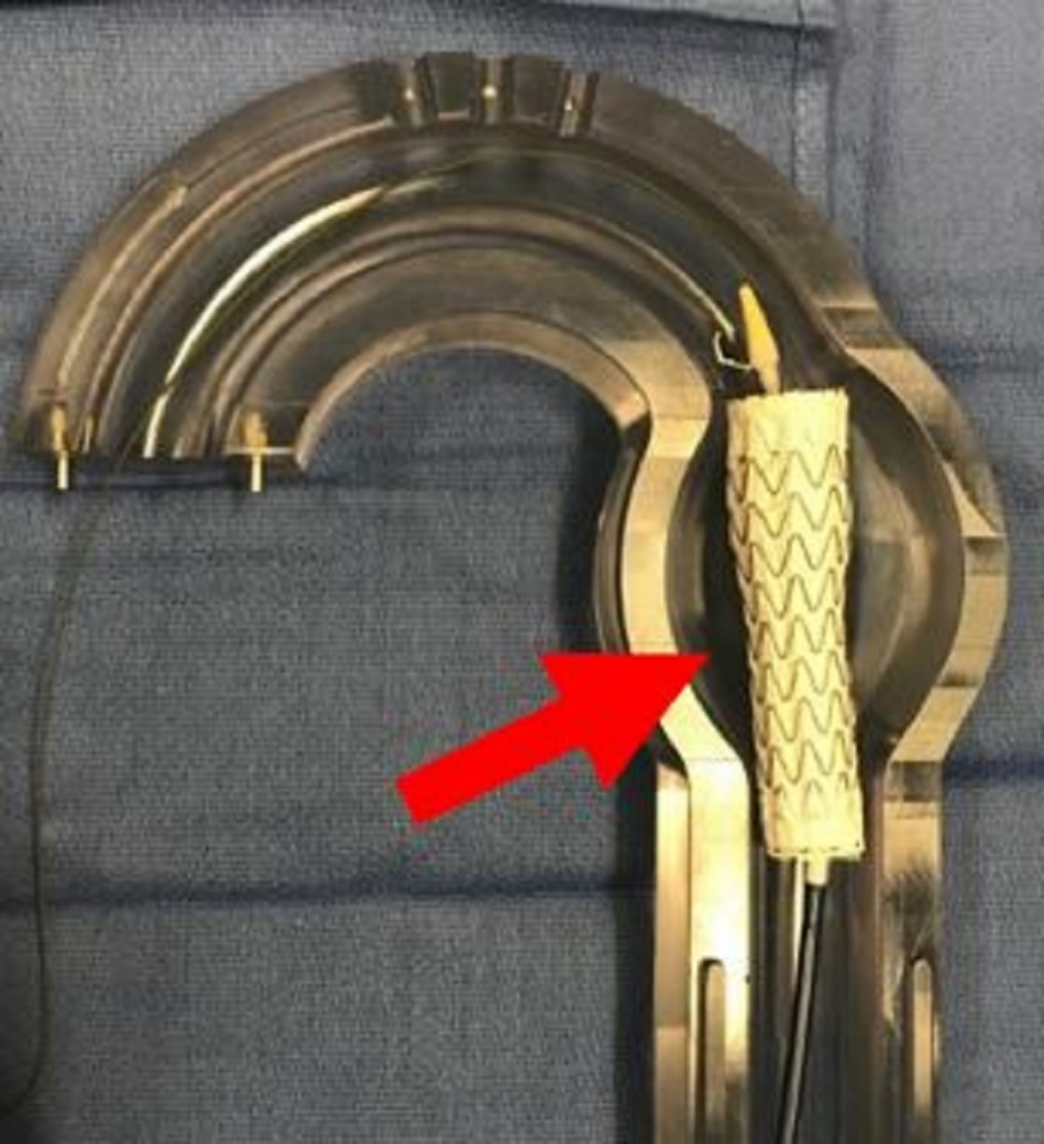
Aortic glass model with anchor thoracic aortic graft deployed (red arrow).

**Figure 7:**
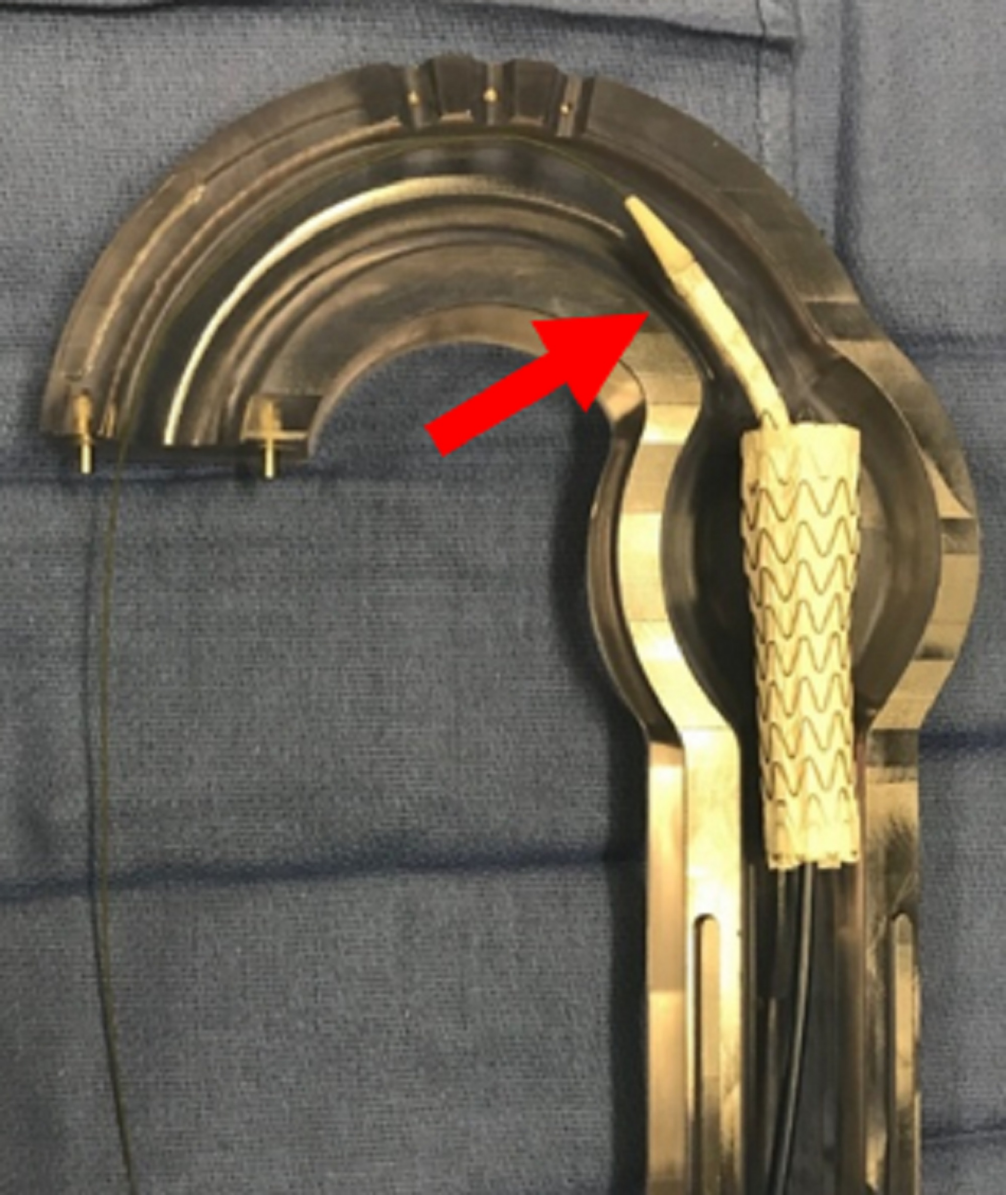
Aortic glass model with pre-deployment (red arrow) of second thoracic aortic graft to build rigidity.

**Figure 8:**
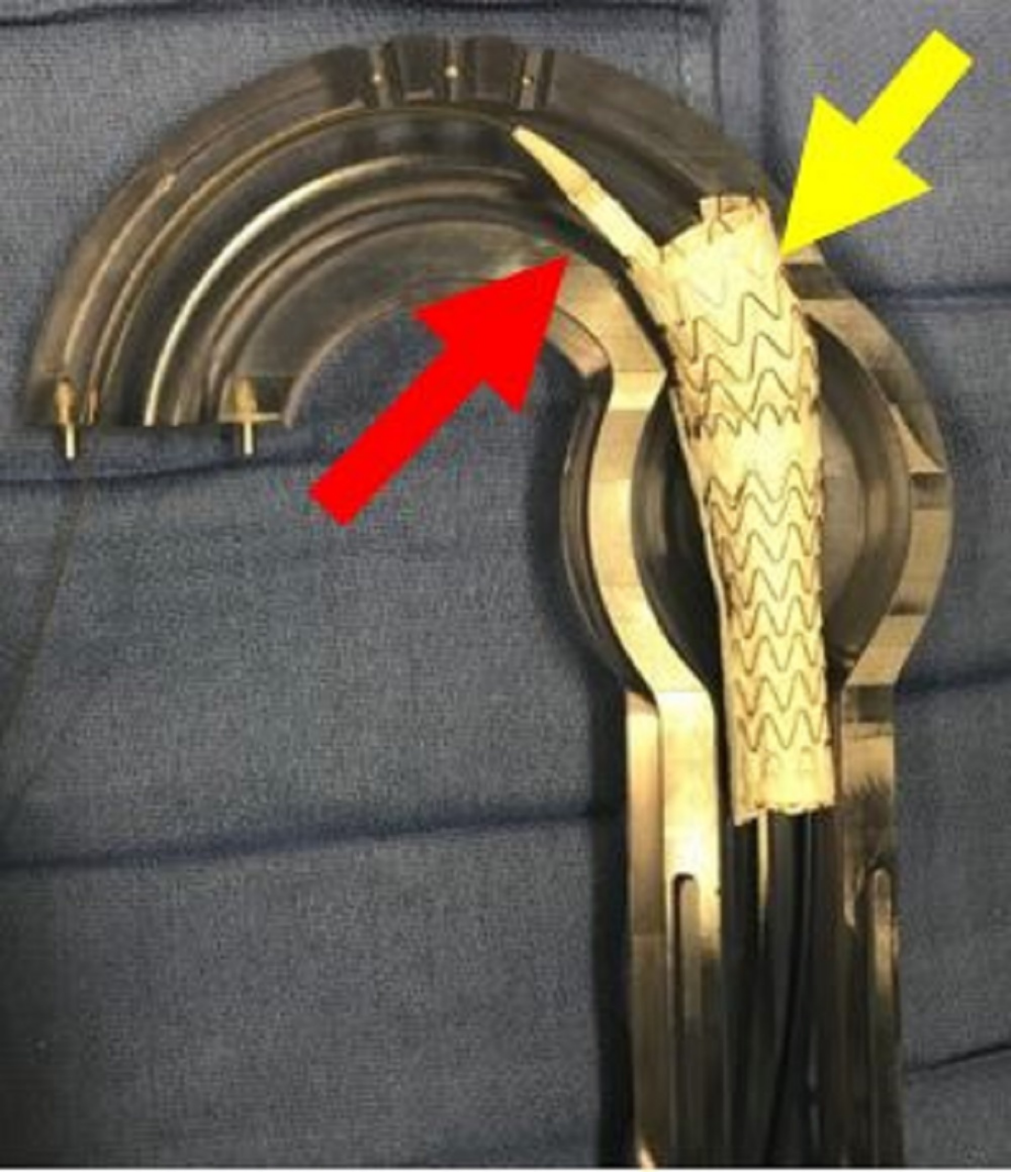
Aortic glass model with deployment of second thoracic aortic graft (yellow arrow) and predeployment placement of third thoracic aortic graft (red arrow). As each graft is placed the amount of overlap increases.

**Figure 9:**
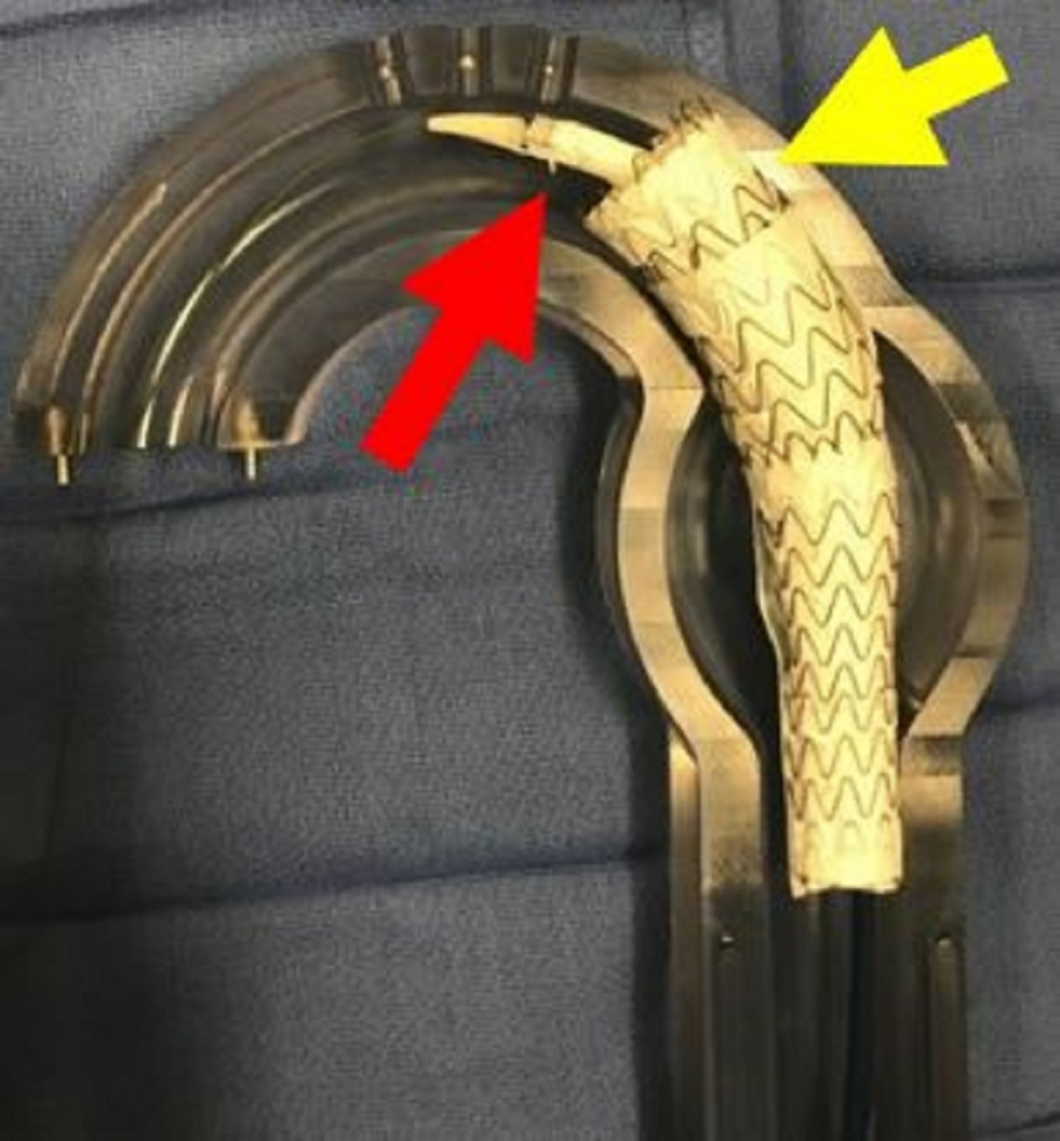
Aortic glass model with deployment of third thoracic aortic graft (yellow arrow) and predeployment placement of final thoracic aortic graft (red arrow) which is the turtlehead portion for conformability.

**Figure 10:**
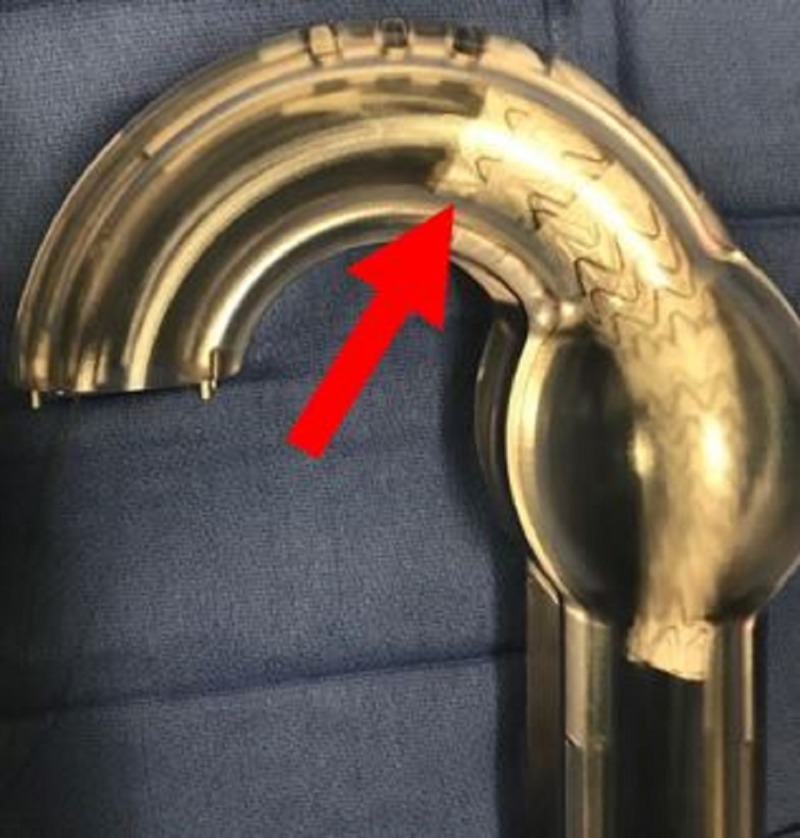
Aortic glass model with deployment of the final thoracic aortic graft (red arrow).

No endoleak or bird-beaking was observed. At this time, angiography of the left subclavian and vertebral arteries was performed. A 14 mm Amplatzer vascular plug (Abbott, St. Paul, MN, USA) was deployed at the origin of the left subclavian artery with preservation of the left vertebral artery. The left brachial arterial access site was closed using manual pressure. The vascular surgical team then surgically closed the right common femoral artery access site and groin region. A glass model was used for additional explanation of the technique (Figures 5-10).

**Figure 11:**
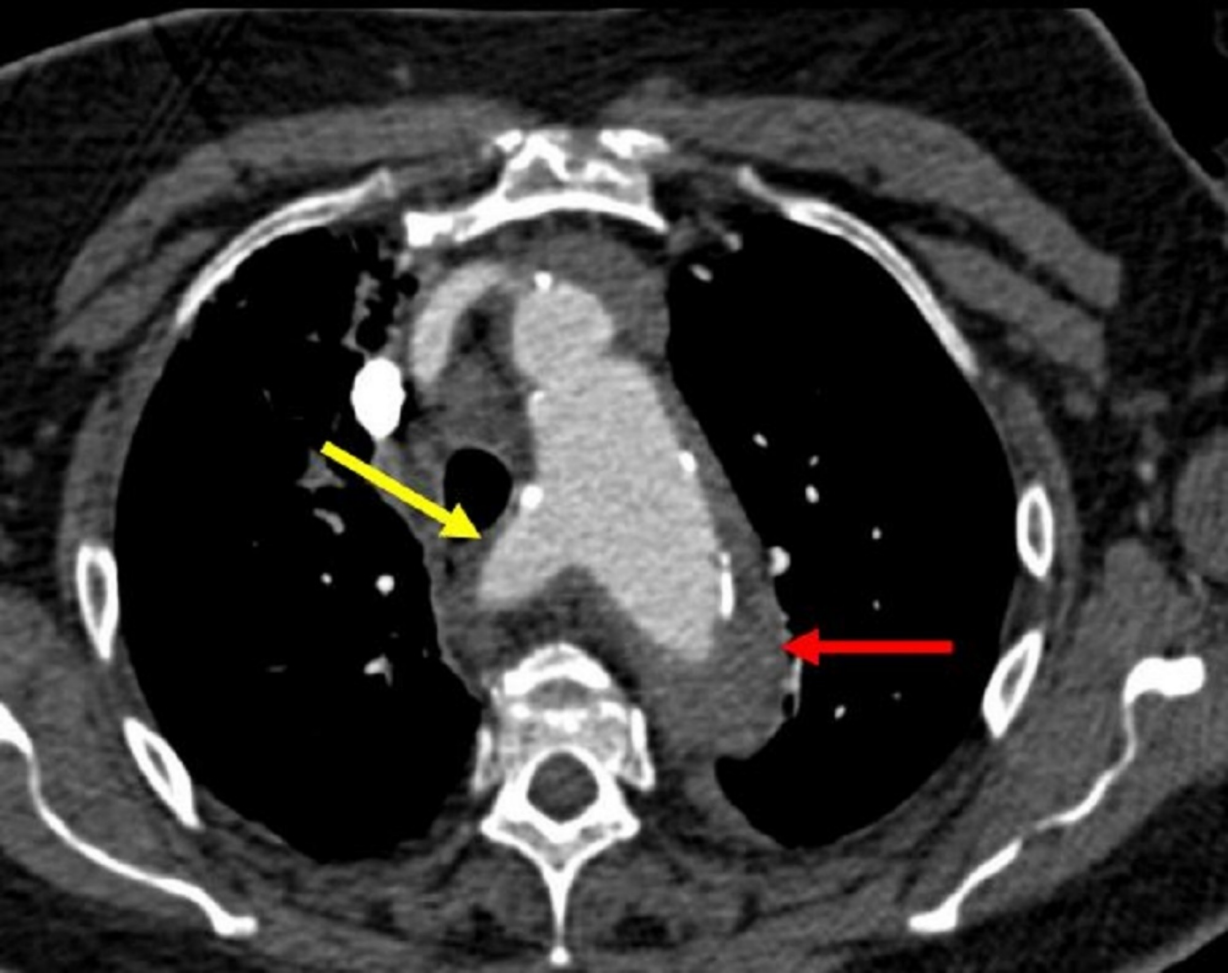
Aberrant right subclavian artery (yellow arrow) and the aortic intramural hematoma (red arrow).

**Figure 12:**
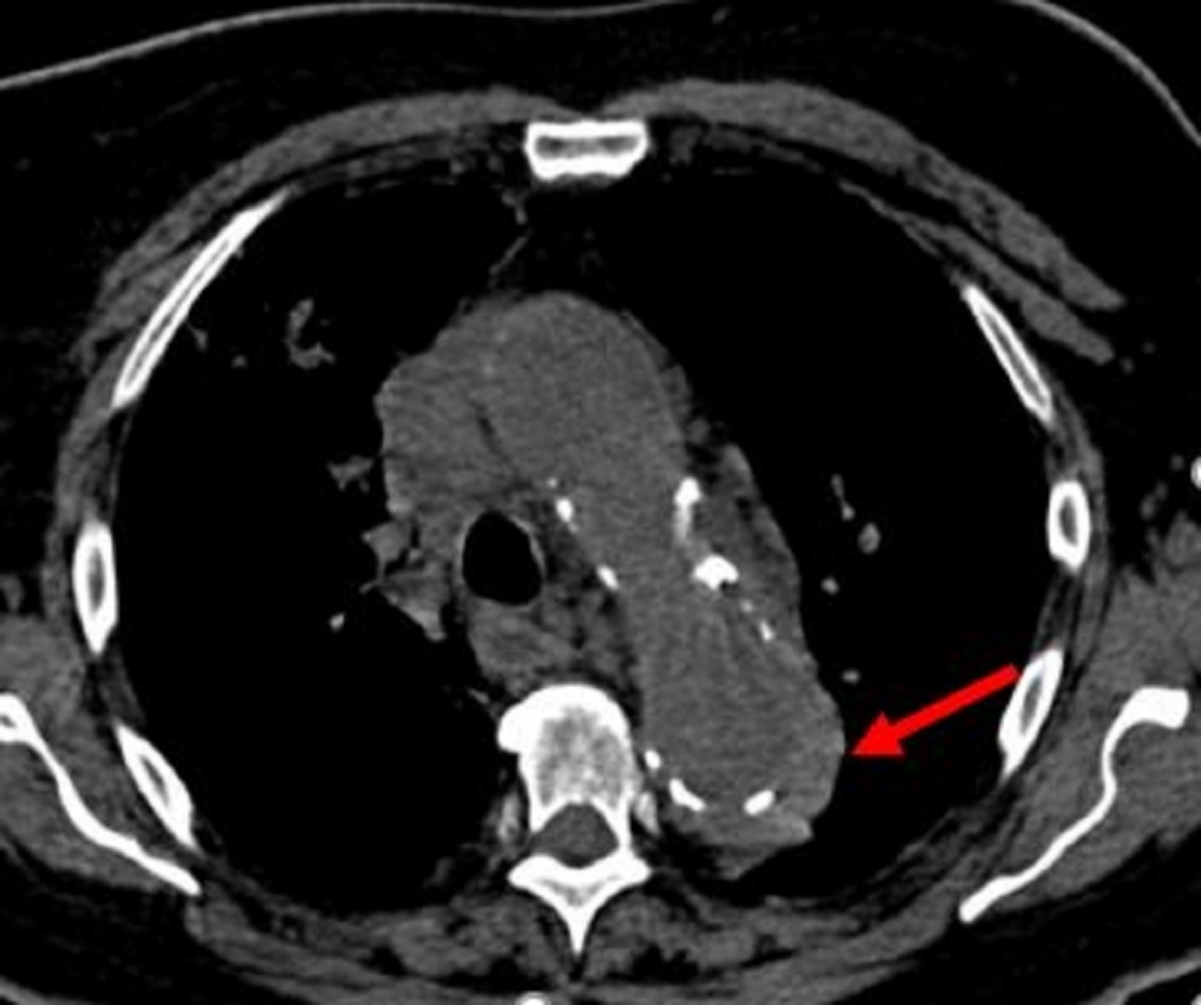
Non-contrast enhanced axial computed tomography at the level of the aortic arch and demonstrates a hyperdense ring around the aorta (red arrow) representing an intramural hematoma.

**Figure 13:**
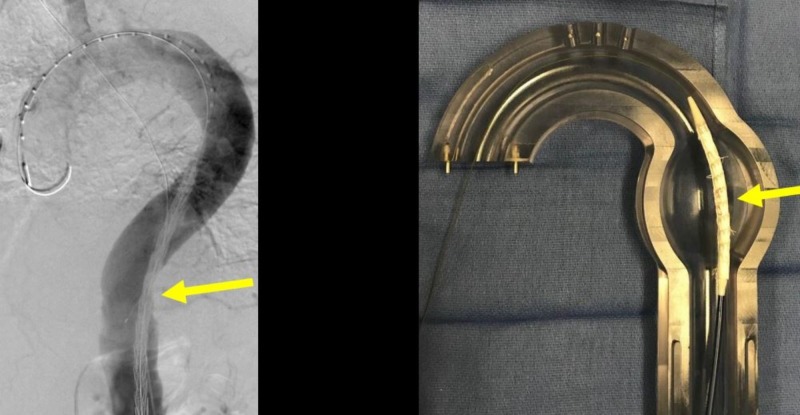
Pre-deployment anchor thoracic aortic graft (yellow arrow) in initial position in the angiogram (left image) and the corresponding glass model (right image).

**Figure 14:**
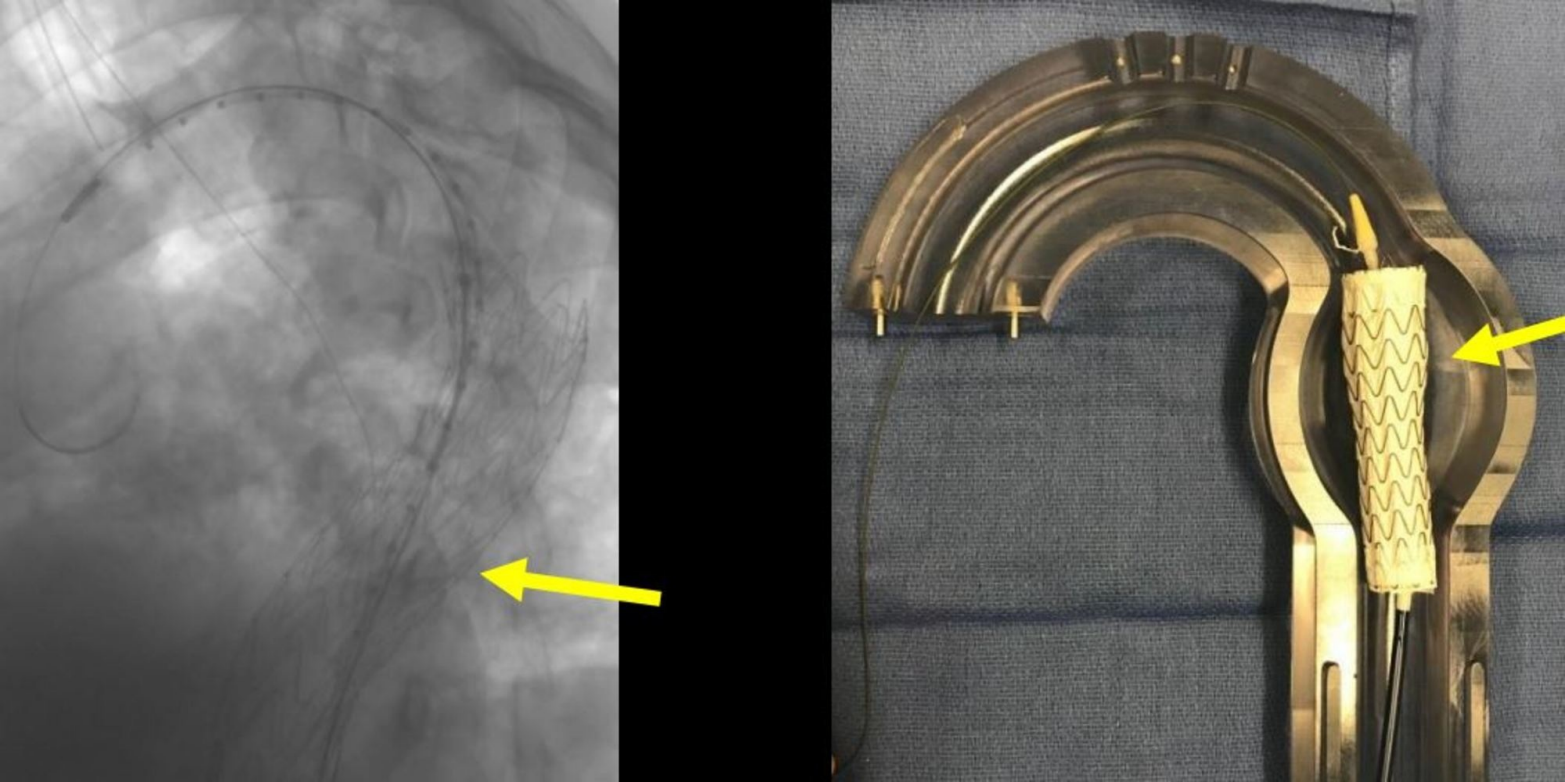
Deployment of the thoracic aortic graft in the angiogram (left image, yellow arrow) and the corresponding glass model (right image).

**Figure 15:**
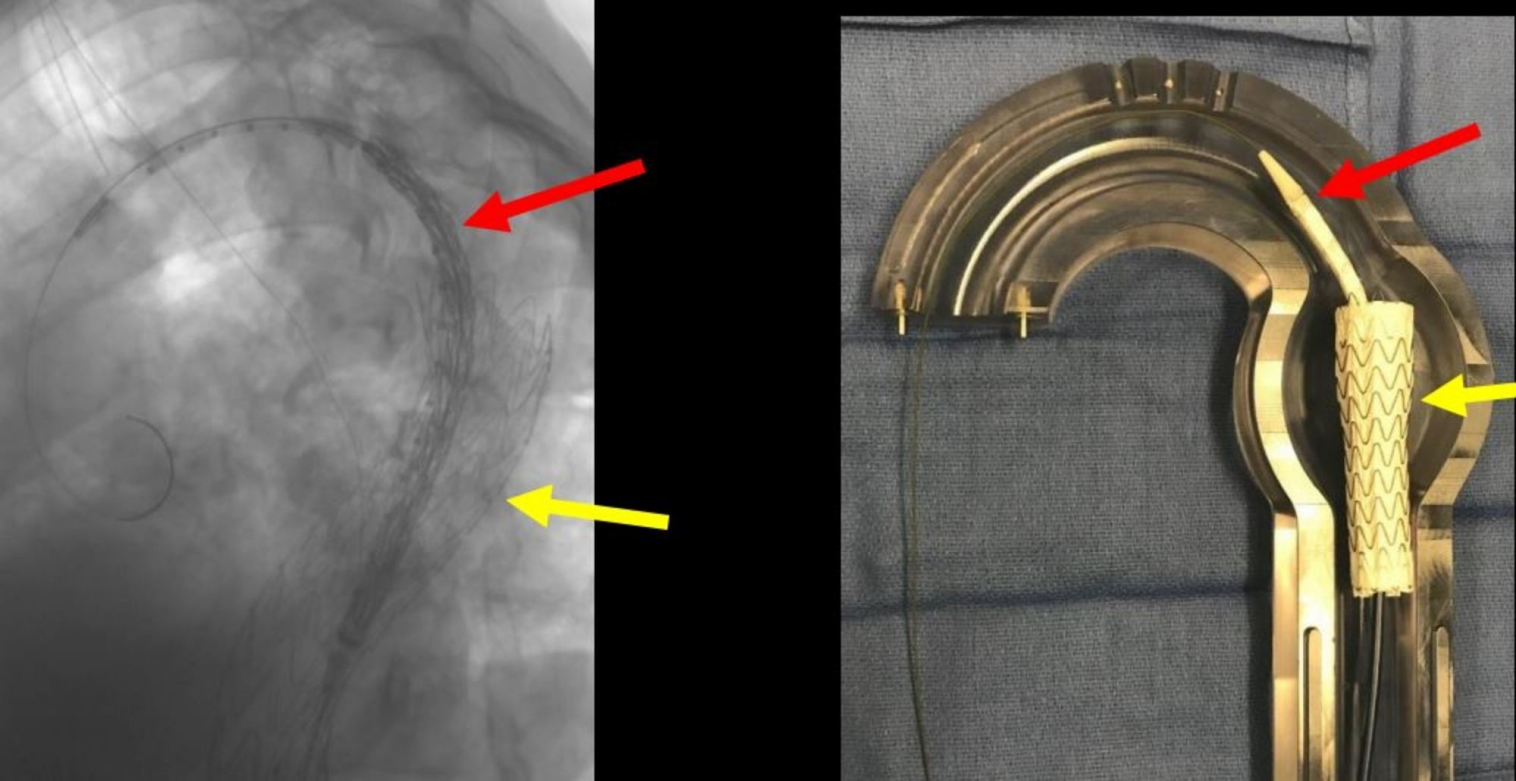
Pre-deployed second thoracic aortic graft (red arrow) and the deployed anchor thoracic aortic graft (yellow arrow) and the corresponding glass model (right image).

**Figure 16:**
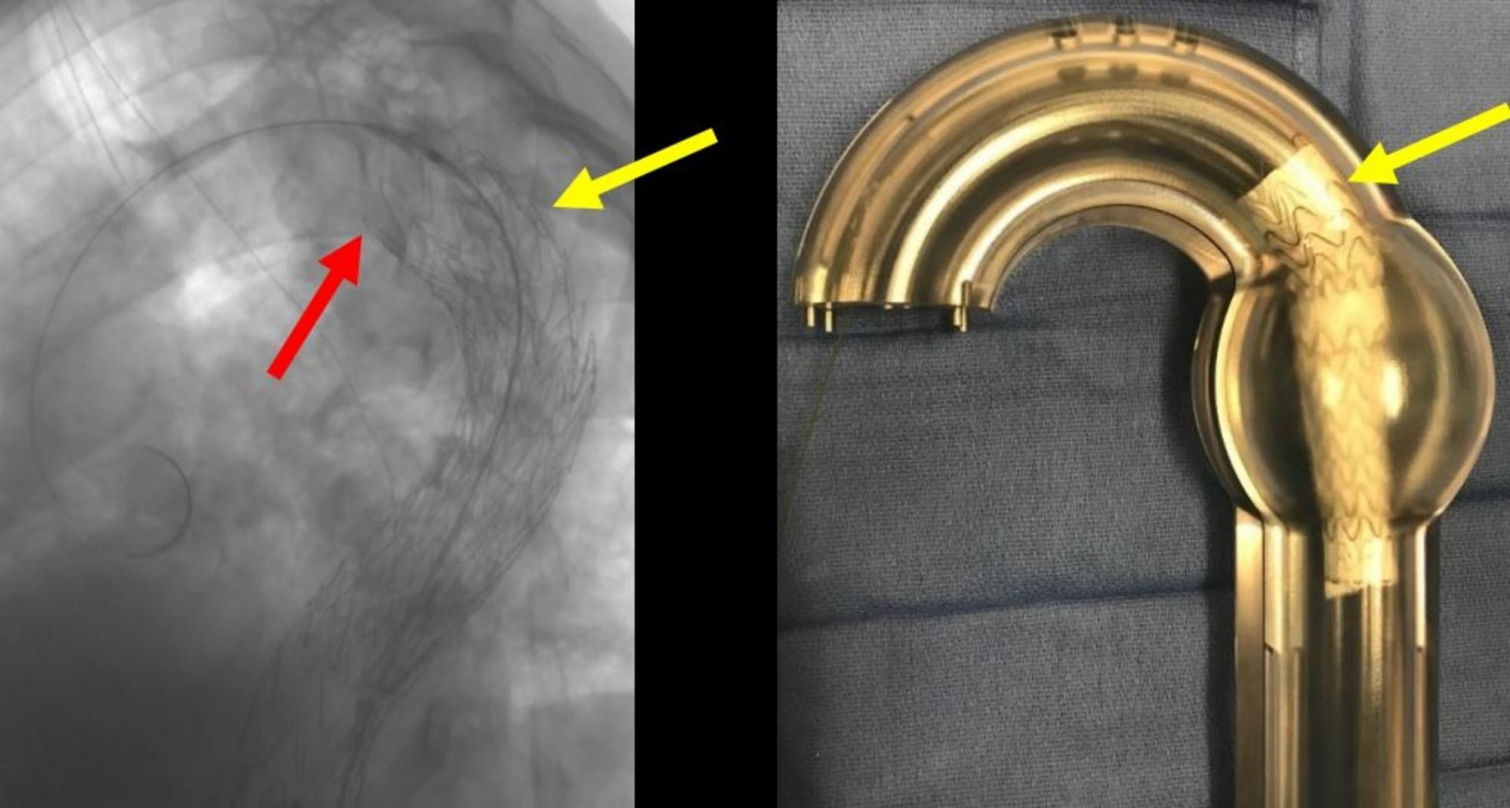
Deployed second thoracic aortic graft (yellow arrow, left image) and the corresponding glass model (right image).

**Figure 17:**
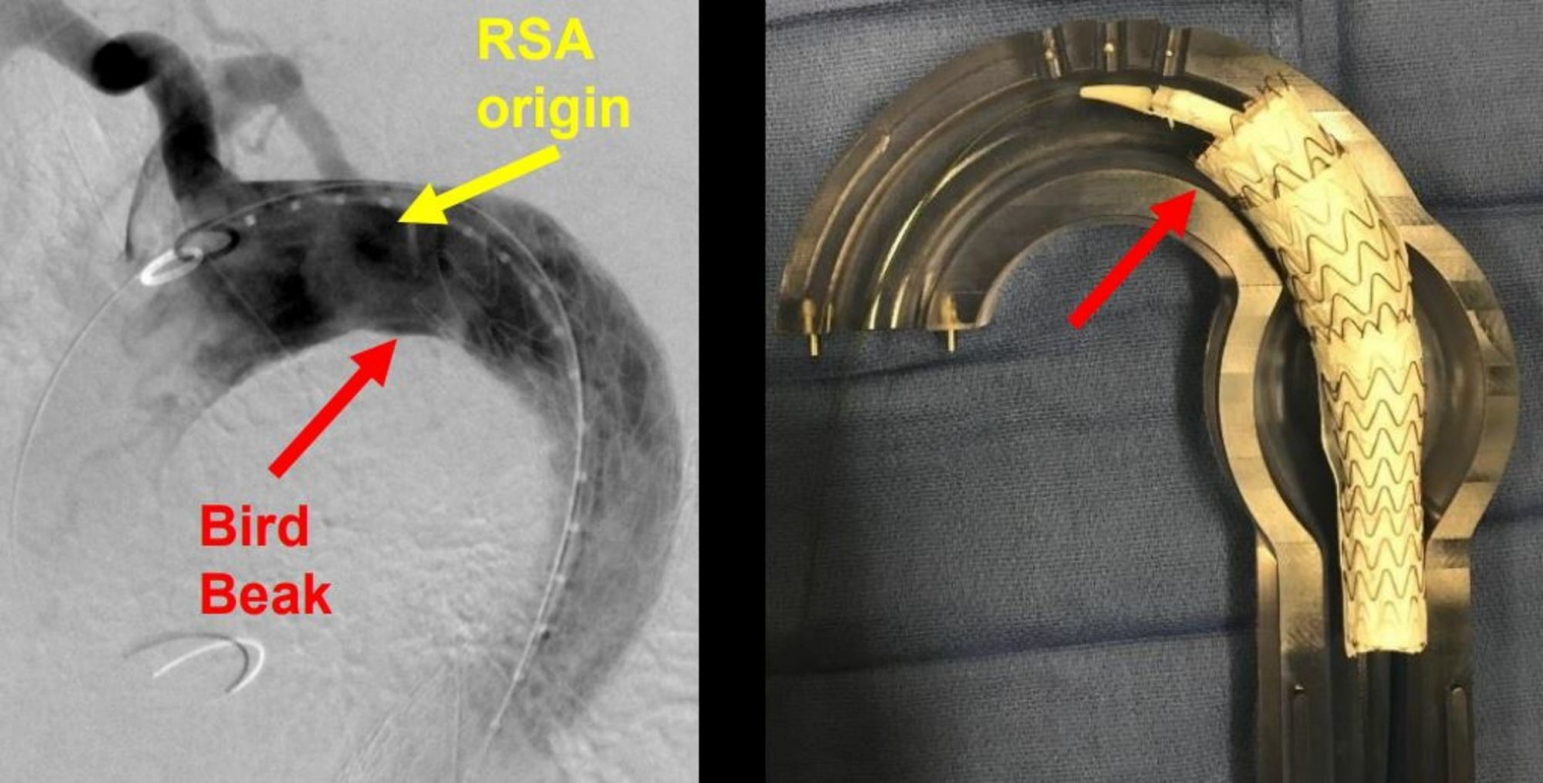
Bird-beaking (red arrow) from the second thoracic aortic graft (left image) and the corresponding glass model (right image). The double density (yellow arrow, left image) delineates the origin of the right subclavian artery (RSA).

**Figure 18:**
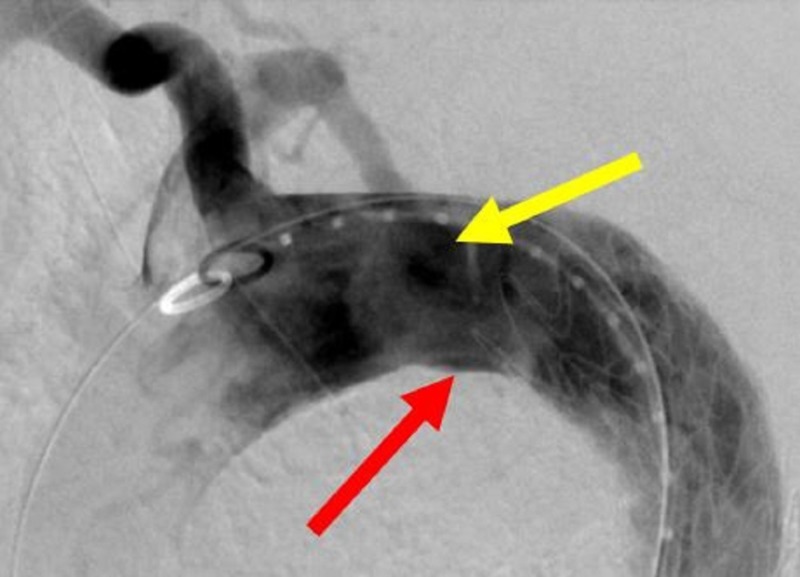
A magnification of the bird-beak (red arrow). The yellow arrow shows the origin of the aberrant right subclavian artery which is to be preserved and not covered.

**Figure 19:**
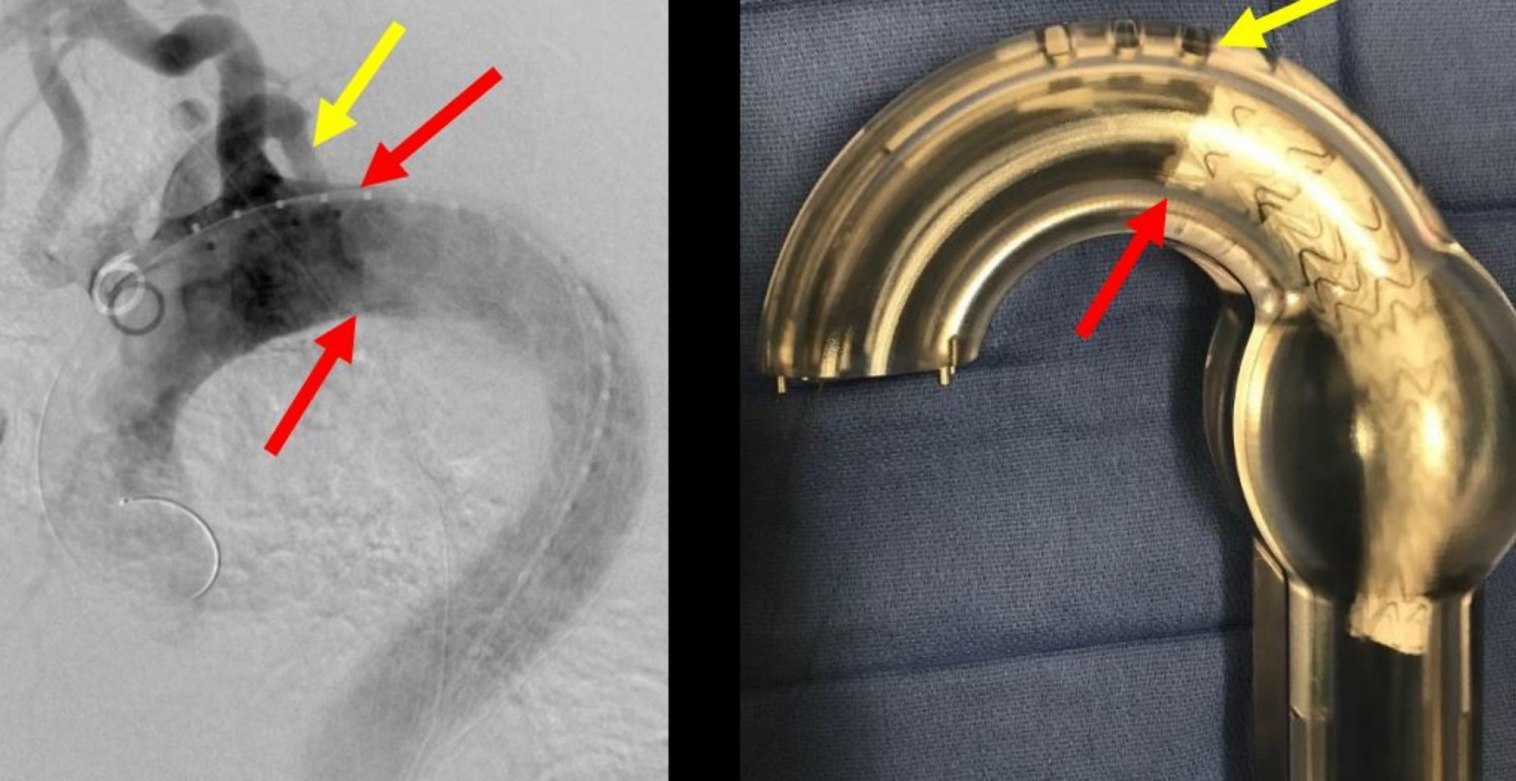
Preserved aberrant right subclavian artery (yellow arrow) after deployment of the third thoracic aortic graft (red arrows) which eliminates the bird-beak and smoothly conforms to the aortic arch in the angiogram (left image) and the corresponding glass model (right image).

**Figure 20:**
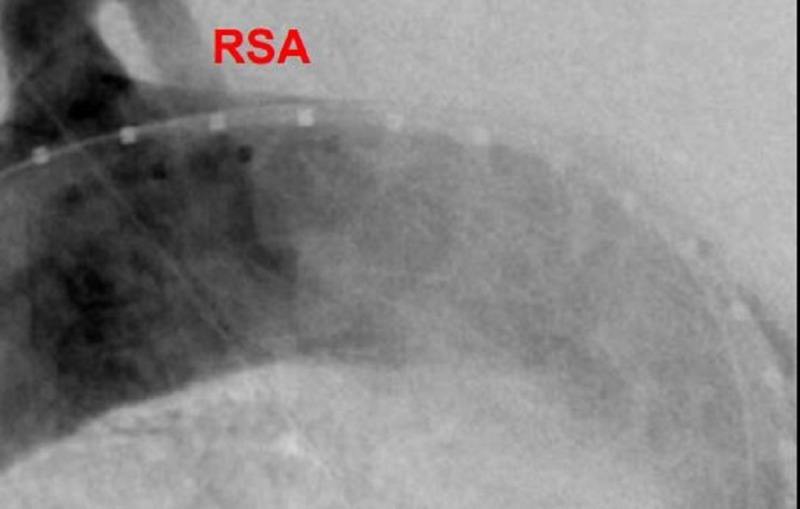
Post-deployment angiogram of the third graft in the turtlehead fashion for a seamless repair.

For the intramural hematomas (IMH) case presented, the aorta diameter measures 37 mm just distal to the aberrant right subclavian artery and 32 mm at the level of the celiac artery. The initial graft placed was 40 mm x 15 cm and landed just above the celiac artery in order to begin the foundation for subsequent support. The second graft, which was placed for rigidity and conformity around the aortic arch was 40 mm x 15 cm. This graft was deliberately placed just short of the target to allow realestate for the final turtlehead device. A significant bird-beak was observed. Because of the nature of the disease process being an IMH, angioplasty was contraindicated. The final graft (40 mm x 10 cm) known as the turtlehead was precisely placed uneventfully just distal to the aberrant right subclavian artery orifice. This was placed with approximately one centimeter of graft material exposed. This provided circumferential wall apposition which completely eliminated the previously seen bird-beak (Figures 11-20).

**Figure 21:**
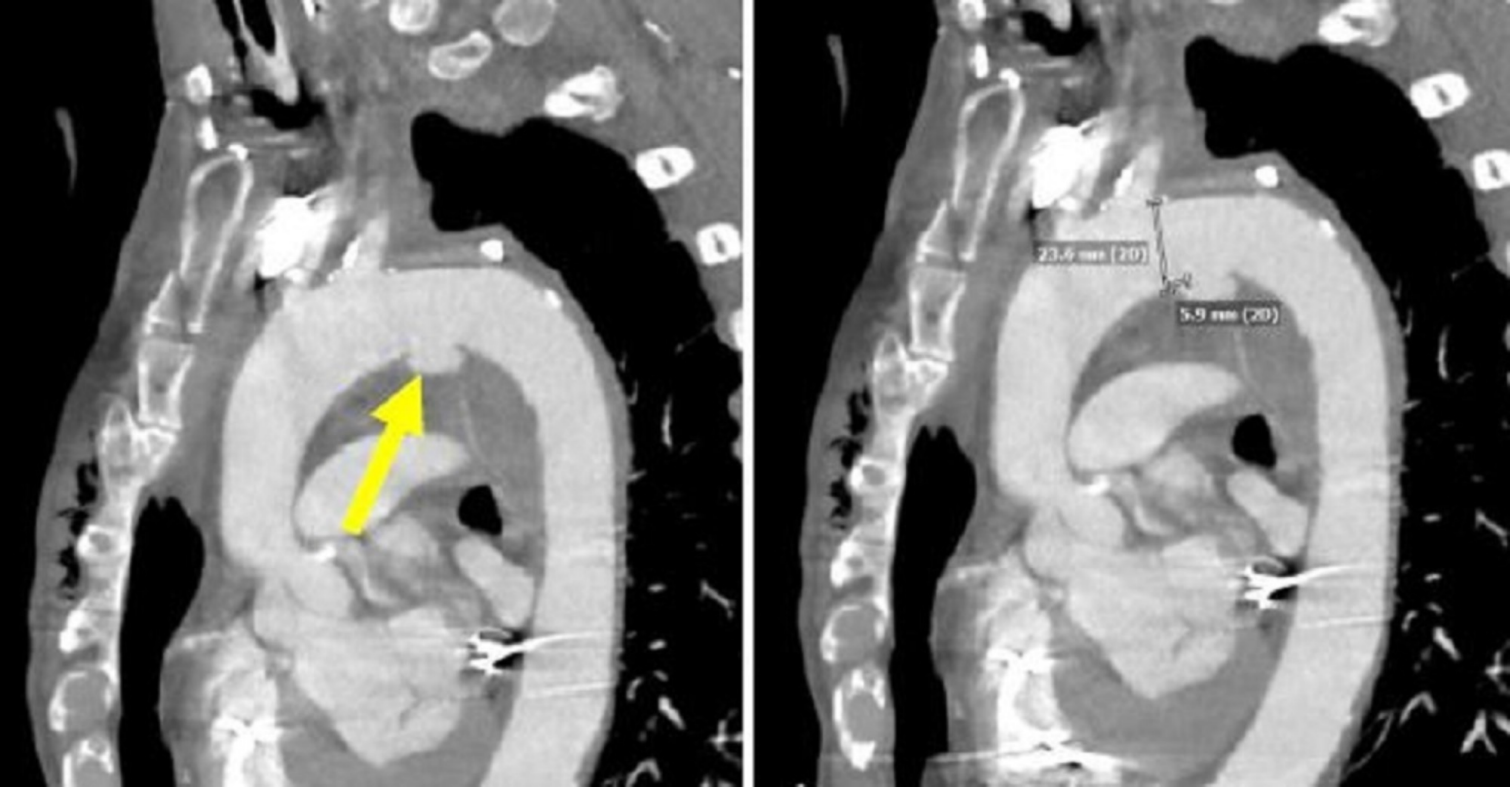
Sagittal computed tomography angiography on the left showing a traumatic pseudoaneurysm (yellow arrow). The image on the right shows the caliper along the lesser curve measuring 5.9 mm as a proximal landing zone neck.

**Figure 22:**
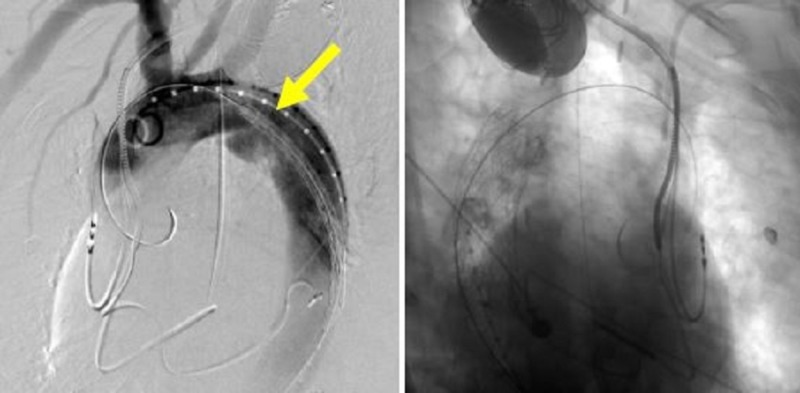
Thoracic aortic angiogram showing the first thoracic aortic graft in place and the proximity to the left subclavian artery on the left image (yellow arrow). The right image shows the first thoracic aortic graft deployed within the aorta.

**Figure 23:**
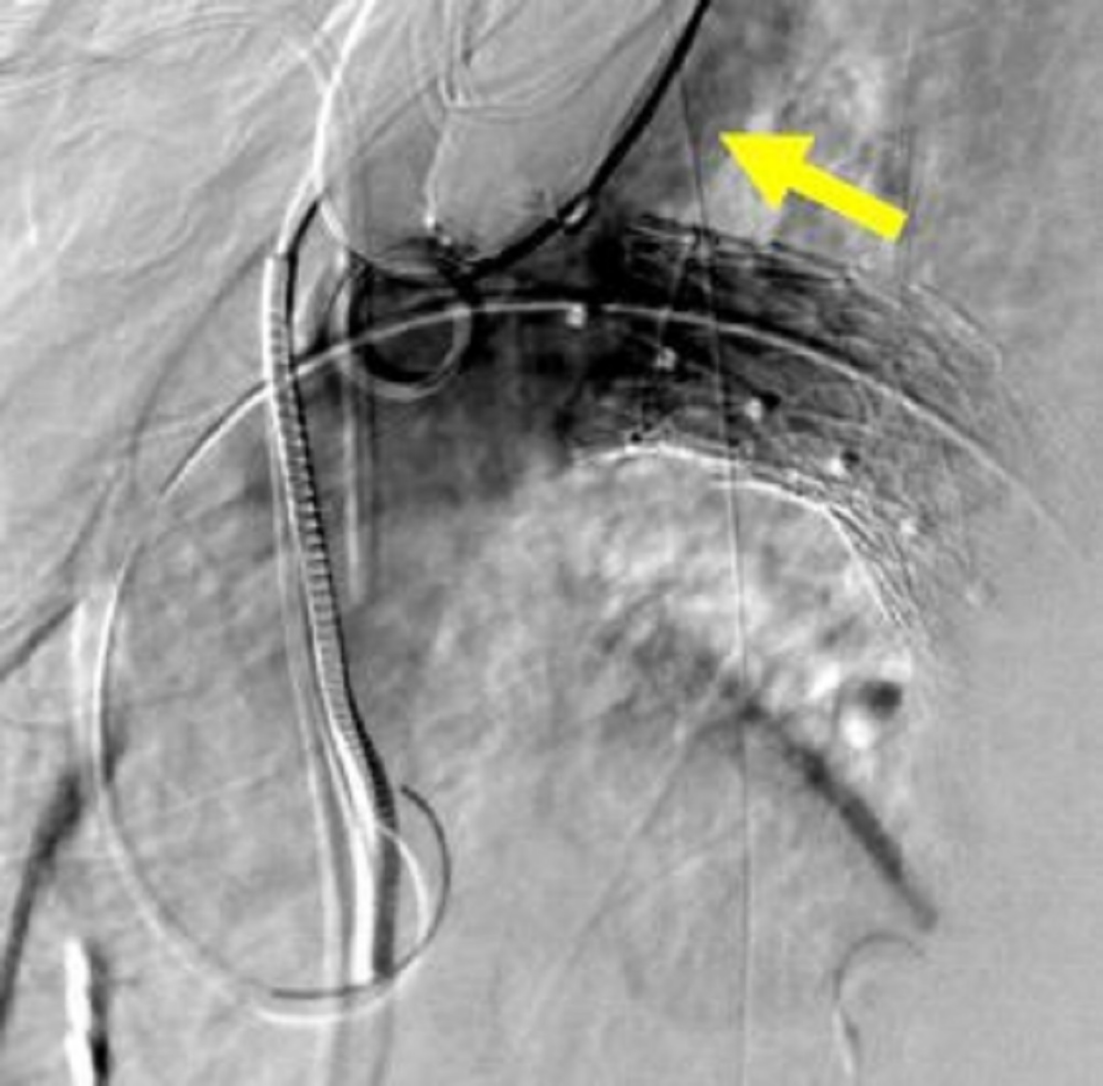
Final thoracic aortic angiogram showing precise exclusion of the traumatic aortic injury and preservation of the left subclavian artery (yellow arrow).

For the trauma case presented, the aorta diameter measures 24 mm just distal to the left subclavian artery (LSA). There is a very short proximal landing zone neck from LSA to the arotic injury. The initial graft placed was 26 mm x 10 cm (W L Gore and Associates, Flagstaff, AZ, USA) and landed approximately 10 mm from the LSA in order to begin the foundation for subsequent support. This graft was deliberately placed just short of the target to allow real-estate for the final turtlehead device. The second and final turtlehead graft, which was placed for precision, was a 28 mm x 10 cm (W L Gore and Associates, Flagstaff, AZ, USA). Angioplasty was contraindicated in this traumatic injury case. Final angiogram showed excellent results with exclusion of the aortic tear and no bird beak (Figures 21-23).

## Discussion

The management and treatment of aortic pathology has undergone significant changes in recent years with the advent of less invasive techniques. Preliminary data has shown less postprocedural complications and mortality with endograft repair in comparison to open repair [7]. Nationwide, more institutions are therefore favoring endovascular repair as first-line option for TAA treatment. Approximately 14% of patients with TAAs greater than 6 cm in diameter have annual risk of rupture or dissection [8]. Therefore, when the aneurysm size is greater than 6.5 cm in an average risk patient, operative repair is recommended [4]. Repair is also recommended if the aneurysm is growing more than 1 cm per year. TAA can be treated with open surgical repair or with endovascular stent grafting. The 30-day mortality of endovascular repair of an unruptured TAA is 5.2% compared to 12% with open surgical repair [9]. Overall, including complication rates and post-treatment re-intervention rates, the endovascular stent grafting of TAA is cost-effective in comparison to the open repair [7]. Therefore, endovascular repair is the favored treatment option in our patients. Traditionally with endovascular approach, a single body graft is utilized the majority of the time. However, in our patients it was not possible to use a single graft due to complex arch anatomy and short landing zones. Using a single graft in our cases could have led to stent migration and bird-beaking. With type 3 aortic arch anatomy, the endovascular graft repair is complicated by birdbeak phenomena [4]. In this situation, the proximal portion of the graft does not sit flush with the lesser curvature of the aortic arch. Due to high pressure blood flow from the ascending aorta, over time this will lead to invagination of the proximal portion, narrowing of the lumen, and migration of the graft. Type I endoleak, retrograde dissection, and graft collapse may occur due to a lack of apposition of the proximal stent-graft along the inner curvature of the aortic arch [10]. By utilizing multiple stent grafts with the turtlehead technique, a more rigid repair can conform to the aortic arch. This technique decreases the chances of encountering the negative sequelae of the bird-beaking due to precise deployment with good apposition to the lesser curvature of the aortic arch. Commercially available thoracic devices were initially derived from prototypes used years before in the infrarenal aorta [11, 12]. The “kilt technique” was developed in 2006 and describes a technique for unfavorable proximal landing zones of the infrarenal aorta. The authors describe their technique by first placing a cuff proximally within a reverse conical neck and then building downward with the exclusion stent graft device [13]. The turtlehead technique on the other hand, uses the final stent graft as a proximal cuff with 90% overlap for precision and elimination of bird-beaking along the natural curvature of the aortic arch. Due to the rigidity of this endograft construct, the chances of migration are reduced.

IMH is a type of aortic dissection. The vasa vasorum ruptures within the arterial wall causing weakness and impending rupture. Thoracic ballooning should be avoided in patients with severe aortic pathology where there is a substantial risk of causing aortic rupture or creating fenestrations [14]. Therefore, in the second case illustrated in this article, angioplasty was not an option to eliminate the bird-beak and using the turtlehead technique was the only endovascular option available. The mechanics of this technique are reliable because of the dominant amount of overlap within the confined area portion of the previous stent graft. There is no room for the final “turtlehead” graft to move or jump. Therefore, the most proximal portion of the stent graft is allowed to precisely flair open with wall apposition. The technique also eliminates the need to force positive pressure on the super stiff wire to attempt to achieve wall apposition on the outer curve. By placing forward tension on the deployment wire, the chances of unintended deployment movement increases. Although placing multiple grafts for difficult aortic pathology repair increases overall costs of the procedure, there are potential long-term benefits including less complications and compounding costs from re-interventions.

**Figure 24:**
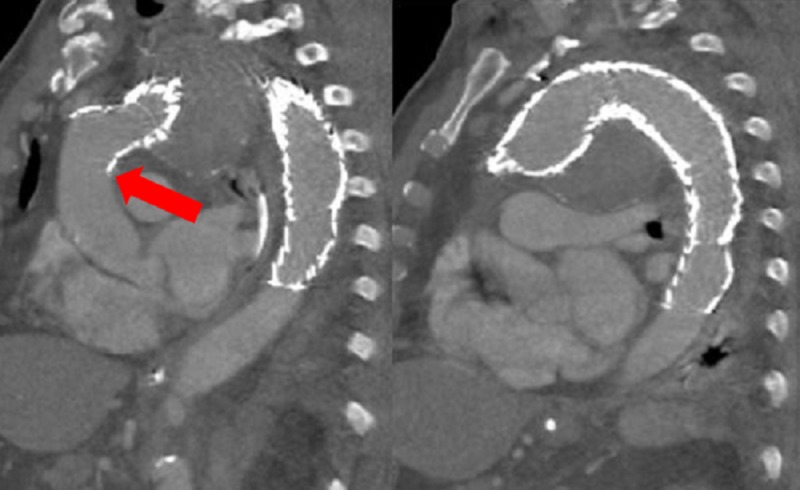
Follow-up imaging sagittal computed tomography of the chest with contrast at one-month post procedure in this patient demonstrated no complications related to stent migration, infolding or endoleak. The left image shows the proximal portion of the thoracic aortic graft (turtlehead) with no bird-beak (red arrow). The right image shows exclusion of the aneurysm with no endoleak.

Follow-up imaging at four-month post procedure of our TAA patient demonstrated no migration with total exclusion of the aneurysm and preservation of the great vessels (Figure 24).

## Conclusions

To date, we have utilized this stent stacking "turtlehead" method for multiple patients without complications related to stent migration, infolding or endoleak. Turtleheading provides an interesting technical strategy to overcome an unfavorable morphological situation at the proximal landing zone. The authors understand the financial burden of this technique and the cost of using multiple endografts. This technique offers a special niche for repair when the aortic arch anatomy is not conducive to other standard repair methods.
